# *Staphylococcus haemolyticus* Infection Modifies Bacterial and Microeukaryotic Gut Communities in Nile Tilapia

**DOI:** 10.3390/ani16192991

**Published:** 2026-09-23

**Authors:** Jesús Salvador Olivier Guirado-Flores, Marcel Martínez-Porchas, Estefanía Garibay-Valdez, Francisco Vargas-Albores, Diana Medina-Félix, Luis Rafael Martínez-Córdova, Francesco Cicala

**Affiliations:** 1 Centro de Investigación en Alimentación y Desarrollo, A.C., Hermosillo 83304, Sonora, Mexico; jguirado223@estudiantes.ciad.mx (J.S.O.G.-F.); estefania.garibay@ciad.mx (E.G.-V.); fvalbores@ciad.mx (F.V.-A.); diana.medina@ues.mx (D.M.-F.); 2 Departamento de Ecología, Universidad Estatal de Sonora, Hermosillo 83100, Sonora, Mexico; 3 Departamento de Investigaciones Científicas y Tecnológicas de la Universidad de Sonora, Universidad de Sonora, Hermosillo 83000, Sonora, Mexico; luis.martinez@unison.mx; 4 Water Research Institute, National Research Council, 28922 Verbania, Italy; fracicala@gmail.com

**Keywords:** *Oreochromis niloticus*, intestinal microbiota, 16S rRNA, 18S rRNA

## Abstract

The fish gut microbiota includes bacterial and microeukaryotic microorganisms that contribute to host nutrition, immune regulation, and intestinal homeostasis. However, the simultaneous responses of both microbial fractions to bacterial infection remain poorly explored in aquaculture species. This study evaluated intestinal microbiota differences associated with experimental *Staphylococcus haemolyticus* infection in Nile tilapia (*Oreochromis niloticus*). Juvenile female tilapia were randomly assigned to either a non-infected control group or a group experimentally infected with *S. haemolyticus*. Intestinal contents were collected seven days post-challenge, and microbial communities were characterized by amplicon sequencing targeting the V3–V4 region of the 16S rRNA gene and the V9 region of the 18S rRNA gene. In the bacterial fraction, infected fish showed higher relative abundances of Proteobacteria and Firmicutes and lower relative abundances of Fusobacteriota, Patescibacteria, and Actinobacteriota. At the genus level, infected fish showed higher relative abundances of *Staphylococcus*, *Mycobacterium*, and other potentially opportunistic bacteria, along with lower relative abundances of *Cetobacterium* and *Romboutsia*. In the microeukaryotic fraction, infected fish showed higher relative abundances of Ciliophora and Ascomycota and a lower relative abundance of Nematozoa. Shannon and Simpson alpha diversity indices were significantly higher in infected fish in both microbial fractions. PICRUSt2-predicted MetaCyc profiles differed between treatments. Control fish showed higher predicted abundances of pathways related to fermentation and vitamin and cofactor metabolism, whereas infected fish showed higher predicted abundances of pathways related to bacterial cell wall metabolism, arginine and polyamine biosynthesis, lipid metabolism, and microbial adaptation. Overall, experimental *S. haemolyticus* infection was associated with taxon-specific differences in intestinal bacterial and microeukaryotic communities and in predicted bacterial functional potential.

## Simple Summary

Fish farming is essential for food production, but bacterial diseases can weaken fish, reduce productivity, and cause economic losses. The intestine of fish contains a diverse community of microorganisms, including bacteria, fungi, and other microscopic organisms, that contribute to digestion, immune defense, and intestinal balance. This study evaluated how infection with *Staphylococcus haemolyticus* affects this intestinal community in Nile tilapia. Infected fish showed differences in selected bacterial and microeukaryotic groups. Some groups commonly linked to intestinal imbalance and opportunistic behavior increased, while others associated with a more stable intestinal environment decreased. The infection also altered the predicted functional potential of the bacterial community, suggesting shifts in nutrient use, vitamin metabolism, cell structure, and adaptation to stress. These findings show that bacterial infection affects the intestine as an interconnected ecosystem.

## 1. Introduction

The fish gut microbiota comprises a complex community of microorganisms, including bacteria, archaea, fungi, protists, and other microscopic eukaryotes. This community participates in essential host processes, such as nutrient metabolism, absorption of dietary compounds, development of the digestive tract, immune regulation, and protection against opportunistic or pathogenic microorganisms [1,2,3]. Therefore, gut microbiota stability is considered an important component of physiological homeostasis and fish health.

Although most studies on fish gut microbiota have focused on the bacterial fraction, the intestine also harbors eukaryotic microorganisms that may contribute to the ecological dynamics of the intestinal ecosystem. These microeukaryotes may include fungi, protists, microscopic metazoans, parasites, and transient organisms derived from food or the aquatic environment [4]. Their interactions with the host and other microorganisms may range from parasitic relationships to commensal, mutualistic, or trophic associations [5,6]. However, in fish, this component remains less explored than the bacterial microbiota, limiting a comprehensive understanding of the intestine as a multi-kingdom ecosystem.

The composition of the gut microbiota can be influenced by environmental and husbandry factors, such as temperature, dissolved oxygen, pH, salinity, diet, and pathogen exposure, as well as by host-related factors, including developmental stage and immune status [7]. When these communities lose their ecological balance, a state of dysbiosis may develop, characterized by the expansion of opportunistic or potentially harmful microorganisms and the reduction in taxa associated with intestinal stability or beneficial functions [8]. In fish, infection-associated microbiota disruption has been reported in different models, including largemouth bronze gudgeon (*Coreius guichenoti*) with furunculosis, zebrafish (*Danio rerio*) infected with *Streptococcus agalactiae*, and Nile tilapia (*Oreochromis niloticus*) infected with *Staphylococcus haemolyticus* or *Providencia vermicola* [9,10,11].

Staphylococcal diseases have been documented in cultured tilapia, demonstrating the pathogenic potential of members of this genus in aquaculture. *Staphylococcus epidermidis* has been associated with natural mass mortality events, and its pathogenicity has been confirmed through experimental challenges that produced ulcerative disease and mortality [12,13]. Within this genus, *Staphylococcus haemolyticus* has recently been identified as a potential pathogen of Nile tilapia. It was isolated from diseased farmed fish exhibiting lethargy, anorexia, erratic swimming, cutaneous nodules and ulcerations, and alterations in internal organs. Experimental infection with a farm-derived isolate reproduced clinical alterations and caused 45% mortality [14]. Although species-specific estimates of its prevalence, outbreak frequency, and production losses are not currently available, its occurrence in diseased farmed fish and its experimentally demonstrated pathogenicity support its consideration as a potentially emerging bacterial pathogen in tilapia aquaculture.

Despite growing evidence of infection-associated variation in the fish gut microbiota, it remains unclear whether bacterial and microeukaryotic communities respond concurrently during infection, particularly in Nile tilapia infected with *S. haemolyticus*. We hypothesized that experimental infection status would be associated with coordinated differences in the bacterial and microeukaryotic fractions of the intestinal microbiota, reflected in community structure, taxonomic composition, diversity patterns, and interkingdom associations. Accordingly, this study evaluated infection-associated differences in the structure, diversity, and taxonomic composition of bacterial and microeukaryotic intestinal communities, as well as in the predicted functional potential of the bacterial community and bacteria–microeukaryote associations in Nile tilapia experimentally infected with *S. haemolyticus*.

## 2. Materials and Methods

### 2.1. Ethical Statement, Fish Origin and Acclimation

Juvenile female Nile tilapia (*Oreochromis niloticus*), certified as pathogen-free, were obtained from the Aquaculture Center of the State of Sonora (CAES, Ciudad Obregón, Sonora, Mexico). Fish were transported to the Wet Laboratory of the Department of Scientific and Technological Research (DICTUS) at the University of Sonora, Hermosillo, Sonora, Mexico, where the experimental trial was conducted. All procedures involving fish maintenance, bacterial challenge, handling, and sampling were performed following the guidelines of the Ethics Committee of the Centro de Investigación en Alimentación y Desarrollo (CIAD, Hermosillo, Sonora, Mexico), under registration number CONBIOÉTICA-26-CEI-001-20200122.

Before the bacterial challenge, fish were acclimated for 15 days in freshwater under controlled laboratory conditions. Temperature was maintained at 20 ± 0.5 °C, and dissolved oxygen was kept above 6.5 mg L^−1^ by continuous aeration. A 12 h light:12 h dark photoperiod was applied throughout the acclimation and experimental periods. Fish were fed twice daily with a commercial diet containing 45% crude protein and 16% lipid, at a daily ration equivalent to 4% of the total biomass (Silver Cup^®^, El Pedregal, Toluca, Mexico).

### 2.2. Experimental Design and Water Quality Monitoring

After acclimation, 48 fish with a mean initial body weight of 21.2 ± 2.8 g were randomly allocated to two experimental treatments: a non-infected control group and a group experimentally infected with *S. haemolyticus*. Each treatment included three replicate aquaria, each with eight fish. The aquaria had a total capacity of 50 L and were maintained with 35 L of freshwater. For microbiota analyses, each aquarium was considered the experimental unit to avoid pseudoreplication.

Water quality was monitored daily during the trial. Ammonium (NH_4_^+^), nitrite (NO_2_^−^), and nitrate (NO_3_^−^) were measured using commercial test kits and an aquaculture photometer (HI 83293, Hanna Instruments, Woonsocket, RI, USA). The recorded water quality values are presented in Supplementary Table S1.

### 2.3. Bacterial Isolate and Inoculum Standardization

The *S. haemolyticus* isolate used in this study was previously recovered from diseased Nile tilapia obtained from aquaculture farms in Chiapas, Mexico, and was subsequently maintained at the Marine Biotechnology Laboratory of the Centro de Investigación en Alimentación y Desarrollo [14]. Identification was based on amplification and bidirectional Sanger sequencing of the nearly complete 16S rRNA gene, comparison of the resulting sequence against GenBank using BLASTN v2.13.0, and maximum-likelihood phylogenetic analysis. Bacterial propagation and quantification were performed according to previously described procedures [11]. Briefly, colony-forming unit (CFU) counts were used to estimate bacterial density, and the suspension was adjusted to a final concentration of 2 × 10^7^ CFU mL^−1^ immediately before inoculation.

### 2.4. Experimental Infection

Fish from the infected treatment were anesthetized by immersion in buffered MS-222 at 50 mg L^−1^ and then intraperitoneally injected with 0.2 mL of the standardized *S. haemolyticus* suspension (2 × 10^7^ CFU mL^−1^) to induce an acute infection. Fish from the control group were anesthetized and injected with 0.2 mL of sterile saline solution via the same route. After inoculation, fish from both treatments were maintained for seven days under the same environmental and feeding conditions described above. During this period, fish were monitored daily for mortality and overt clinical signs.

The inoculum concentration was selected based on a preliminary median lethal dose (LD_50_) assay conducted with *S. haemolyticus* under experimental conditions equivalent to those used in the present study. Fish were intraperitoneally injected with 0.2 mL of bacterial suspensions containing 1 × 10^7^, 1 × 10^8^, or 1 × 10^9^ CFU mL^−1^ and monitored for seven days. Mortality data were transformed to probit values and analyzed against the log_10_-transformed bacterial concentration to estimate the inoculum concentration corresponding to the LD_50_. Based on this estimate, the challenge suspension was standardized to 2 × 10^7^ CFU mL^−1^. Therefore, the selected inoculum represented an acute challenge based on the preliminary LD_50_ estimate.

### 2.5. Intestinal Content Collection

At seven days post-challenge, fish were fasted for 24 h before sampling. Fish were euthanized using an overdose of tricaine methanesulfonate (MS-222; 250 mg L^−1^). The abdominal cavity was opened under aseptic conditions, and the intestinal tract was carefully removed. Intestinal contents were collected using the scraping method and transferred into sterile microcentrifuge tubes [15].

For each aquarium, intestinal contents from three randomly selected surviving fish were pooled to obtain one composite sample per experimental replicate. Thus, three pooled samples were obtained per treatment. Samples were immediately frozen and stored at −80 °C until DNA extraction.

### 2.6. DNA Extraction and Amplicon Sequencing

Total DNA was extracted from 250 mg of pooled intestinal content using the FastDNA™ Spin Kit for Soil (MP Biomedicals, Santa Ana, CA, USA), following the manufacturer’s instructions. Before extraction, samples were mechanically homogenized using a FastPrep-5G instrument (MP Biomedicals, Santa Ana, CA, USA) at 6.0 m s^−1^ for 40 s. Homogenized samples were then centrifuged at 14,000× *g* for 5 min. DNA concentration was measured using a Qubit 3.0 fluorometer (Thermo Fisher Scientific, Waltham, MA, USA), and DNA integrity was evaluated by electrophoresis on a 1% agarose gel.

The bacterial community was characterized by amplification of the V3–V4 region of the 16S rRNA gene using primers 347F (5′-GGAGGCAGCAGTRRGGAAT-3′) and 803R (5′-CTACCRGGGTATCTAATCC-3′) [16]. The intestinal microeukaryotic community was analyzed by amplification of the V9 region of the 18S rRNA gene using primers 1391f (5′-GTACACACCGCCCGTC-3′) and EukBr (5′-TGATCCTTCTGCAGGTTCCACCTAC-3′) [17]. Library preparation and sequencing were carried out at the Arizona Genetics Core, University of Arizona, Tucson, AZ, USA. Amplicon libraries were sequenced on an Illumina MiSeq platform using paired-end chemistry with a 2 × 250 bp configuration. Raw sequence reads were deposited in the NCBI Sequence Read Archive (SRA) under BioProject accession PRJNA1482159, with associated BioSample accessions SAMN61115009–SAMN61115011, SAMN61115015–SAMN61115020, and SAMN61115024–SAMN61115026.

### 2.7. Amplicon Sequence Processing and Taxonomic Assignment

The 16S rRNA and 18S rRNA datasets were processed independently in QIIME 2 version 2025.7. Raw paired-end reads were imported into QIIME 2 and processed with the DADA2 plugin to perform quality filtering, denoising, paired-end read merging, and chimera removal [18]. Trimming and truncation parameters were selected separately for each marker according to the quality profile of the sequencing reads. The resulting amplicon sequence variants (ASVs), representative sequences, and feature tables were retained for downstream analyses. Per-sample input read counts and sequence counts retained in the final filtered feature tables are provided in Supplementary Table S2.

For the 16S rRNA and 18S rRNA datasets, representative ASV sequences were independently aligned with MAFFT and used to construct phylogenetic trees with FastTree using the “qiime phylogeny align-to-tree-mafft-fasttree” pipeline. The resulting rooted trees were used for UniFrac-based beta diversity analyses.

Taxonomic assignment was performed using Naïve Bayes classifiers trained with the SILVA v138 reference database [19]. Marker-specific classifiers were generated for the V3–V4 region of the 16S rRNA gene and the V9 region of the 18S rRNA gene. Reference sequences were filtered according to the target domain, using Bacteria for the 16S rRNA dataset and Eukaryota for the 18S rRNA dataset. Following taxonomic assignment, ASVs classified as Vertebrata were excluded from the 18S rRNA feature table before downstream analyses to minimize the contribution of host-derived sequences. Taxonomic profiles were summarized from phylum to the lowest reliable taxonomic level, and relative abundance tables were generated independently for bacterial and microeukaryotic communities.

### 2.8. Diversity, Differential Abundance and Correlation Analyses

Alpha and beta diversity analyses were performed using MicrobiomeAnalyst 2.0 [20]. Before alpha- and beta-diversity analyses, the final 16S and 18S feature tables were independently rarefied to 18,672 and 5082 sequences per sample, respectively, corresponding to the minimum sequencing depth in each dataset. Alpha diversity was calculated at the ASV level using the Shannon and Simpson indices. For each marker and diversity index, residual normality was evaluated using the Shapiro–Wilk test, and homogeneity of variances was assessed using Levene’s test centered on the median before applying one-way ANOVA. Differences between control fish and fish infected with *S. haemolyticus* were evaluated using one-way ANOVA, with statistical significance set at *p* < 0.05.

Beta diversity was estimated using Bray–Curtis, Jaccard, weighted UniFrac, and unweighted UniFrac distance matrices. Differences in microbial community composition between treatments were evaluated using PERMANOVA, a distance-based multivariate test. Community dissimilarities between treatments were visualized by principal coordinates analysis (PCoA). In addition, the Microbiome Regression-Based Kernel Association Test (MiRKAT) was used as a complementary kernel-based regression approach to test the global association between treatment condition and microbial community composition. Both analyses were performed in MicrobiomeAnalyst 2.0 [20].

For differential abundance analysis, filtered, unrarefied ASV count tables were taxonomically agglomerated at the phylum and genus levels, and differences between treatments were evaluated using DESeq2 implemented in MicrobiomeAnalyst 2.0 [20,21]. Relative abundance tables were used for taxonomic visualization and descriptive comparisons. Pearson correlation analysis was performed using relative abundance data at the phylum level for bacteria and at comparable high-level taxonomic ranks for microeukaryotes. Correlations with *p* < 0.05 were retained for network visualization.

### 2.9. Functional Prediction of the Bacterial Community

The functional potential of the bacterial community was inferred from the 16S rRNA ASV table and representative sequences using PICRUSt2 version 2.5.0 [22]. This analysis was performed only for the bacterial dataset. Representative ASV sequences were placed into the PICRUSt2 prokaryotic reference phylogeny, and 16S rRNA gene copy number was predicted using hidden-state prediction. Enzyme Commission (EC) gene family abundances were then predicted for each ASV.

ASV abundances were normalized by the predicted 16S rRNA gene copy number and combined with predicted EC gene family copy numbers to generate metagenomic functional profiles. MetaCyc pathway abundances were reconstructed from the predicted EC profiles using the MinPath algorithm implemented in the PICRUSt2 pathway pipeline [23,24]. Pathway descriptions were assigned using the MetaCyc pathway description map included in PICRUSt2.

Unstratified MetaCyc pathway abundance tables were used for downstream differential abundance analysis. Differentially abundant predicted MetaCyc pathways between control fish and fish infected with *S. haemolyticus* were identified using edgeR v3.40.0 [25]. Statistical significance was determined using false discovery rate (FDR) correction, with FDR < 0.05 considered significant. Differentially abundant pathways were visualized using a volcano plot based on log_2_ fold change and −log_10_(FDR).

## 3. Results

### 3.1. Mortality Following Experimental Infection

During the seven-day post-challenge period, no mortality was recorded in the control group, whereas cumulative mortality reached 25% in fish challenged with *S. haemolyticus*, corresponding to survival rates of 100% and 75%, respectively. Infected fish also exhibited lethargy and an apparent loss of appetite, whereas these signs were not observed in the control group.

### 3.2. Gut Bacterial Microbiota

#### 3.2.1. Bacterial Relative Abundance

A total of 14 bacterial phyla were identified in the gut microbiota of Nile tilapia. Fusobacteriota was the dominant bacterial phylum, with a mean relative abundance of 76.62 ± 0.62% in control fish and 60.53 ± 4.60% in fish infected with *S. haemolyticus*. Firmicutes represented 12.92 ± 0.15% and 25.71 ± 3.64% of the bacterial community, respectively. Together, these phyla accounted for 89.54 ± 0.53% of the bacterial community in the control group and 86.23 ± 0.96% in the infected group.

Differential abundance analysis using DESeq2 identified significant taxon-specific differences between control fish and fish infected with *S. haemolyticus*. Proteobacteria and Firmicutes had significantly higher relative abundances in infected fish, whereas Patescibacteria, Fusobacteriota, and Actinobacteriota had significantly lower relative abundances (*p* < 0.05) (Figure 1).

At the genus level, *Cetobacterium*, *Iamia*, *Nordella*, *Dietzia*, *Terrimicrobium*, *Romboutsia*, and *Nocardioides* had significantly lower relative abundances in infected fish. In contrast, *Staphylococcus*, *Aurantimicrobium*, *Pseudonocardia*, *Bosea*, *Legionella*, *Gemmobacter*, and *Mycobacterium* had significantly higher relative abundances in infected fish than in control fish (*p* < 0.05) (Figure 2).

#### 3.2.2. Bacterial Alpha Diversity

Alpha diversity of gut bacterial communities was assessed using Shannon and Simpson indices at the ASV level (Figure 3). Both indices revealed significant differences (*p* < 0.05) between control fish and fish infected with *S. haemolyticus*. Specifically, Shannon and Simpson diversity indices were higher in infected fish compared to controls.

#### 3.2.3. Bacterial Beta Diversity

The PCoA plots showed clear clustering patterns, indicating visible separation between control fish and *S. haemolyticus*-infected fish (Figure 4). Detailed PERMANOVA and MiRKAT statistics for each distance metric are provided in Supplementary Table S3. However, the statistical outcomes differed depending on the distance metric and test used. For Bray–Curtis and Jaccard distances, PERMANOVA did not detect significant differences in gut bacterial communities between control fish and infected fish (*p* > 0.05). In contrast, MiRKAT detected significant associations for both Bray–Curtis and Jaccard distances (*p* < 0.05). For unweighted and weighted UniFrac distances, neither PERMANOVA nor MiRKAT detected significant associations between groups (*p* > 0.05).

#### 3.2.4. Predicted MetaCyc Functional Pathways

Differential abundance analysis using edgeR identified predicted MetaCyc pathways that differed between control fish and fish infected with *S. haemolyticus* (Figure 5). Of the 380 pathways evaluated, 256 showed significant differential abundance after FDR correction (FDR < 0.05). Among the significant pathways, those with higher predicted abundance in infected fish were mainly associated with bacterial cell wall metabolism, amino acid and polyamine biosynthesis, nucleotide degradation, lipid biosynthesis, and cofactor-related metabolism. Representative pathways included peptidoglycan biosynthesis in staphylococci, anhydromuropeptide recycling, L-arginine biosynthesis, arginine and polyamine biosynthesis, purine and adenosine nucleotide degradation, fatty acid biosynthesis, oleate and stearate biosynthesis, heme biosynthesis, phylloquinol biosynthesis, and mycolate biosynthesis.

In contrast, pathways with higher predicted abundance in control fish were mainly associated with fermentative metabolism, vitamin and cofactor biosynthesis, carbohydrate-related cell-envelope precursors, and the degradation of aromatic or environmental compounds. Representative pathways included heterolactic fermentation, the Bifidobacterium shunt, biotin biosynthesis, adenosylcobalamin biosynthesis and salvage, GDP-mannose biosynthesis, GDP-D-glycero-α-D-manno-heptose biosynthesis, protocatechuate degradation, toluene degradation, and CMP-3-deoxy-D-manno-octulosonate biosynthesis.

### 3.3. Gut Microeukaryotic Microbiota

#### 3.3.1. Microeukaryotic Relative Abundance

A total of nine microeukaryotic phyla were identified in the gut microbiota of Nile tilapia. Holozoa was the dominant group, representing approximately 60% of the total sequences obtained.

Differential abundance analysis using DESeq2 identified significant taxon-specific differences between control fish and fish infected with *S. haemolyticus*. Ciliophora and Ascomycota had significantly higher relative abundances in infected fish, whereas Nematozoa had a significantly lower relative abundance (*p* < 0.05) (Figure 6).

#### 3.3.2. Microeukaryotic Alpha Diversity

Microeukaryotic alpha diversity was evaluated at the ASV level using the Shannon and Simpson indices. Both indices showed significant differences between control fish and fish infected with *S. haemolyticus* (*p* < 0.05). Shannon and Simpson diversity values were higher in infected fish than in the control group (Figure 7).

#### 3.3.3. Microeukaryotic Beta Diversity

The PCoA plots showed visible clustering patterns, suggesting treatment-related differences in the microeukaryotic community structure between control fish and fish infected with *S. haemolyticus* (Figure 8). Detailed PERMANOVA and MiRKAT statistics for each distance metric are provided in Supplementary Table S3. However, the statistical support for these patterns depended on the distance metric and test used. PERMANOVA and MiRKAT did not detect significant differences using Jaccard, Bray–Curtis, or unweighted UniFrac distances (*p* > 0.05). In contrast, MiRKAT detected significant differences between groups using weighted UniFrac distance (*p* < 0.05), whereas PERMANOVA did not detect significant differences for this metric (*p* > 0.05).

### 3.4. Correlation Analysis Between Bacterial and Microeukaryotic Communities

Pearson correlation analysis revealed significant associations between bacterial and microeukaryotic phyla in the gut microbiota of Nile tilapia (Figure 9). Proteobacteria, Firmicutes, and Bacteroidota were positively correlated with Ciliophora (r = 0.965–0.992, *p* < 0.05) and Ascomycota (r = 0.915–0.961, *p* < 0.05). In contrast, Holozoa, Nematozoa, and Protalveolata were negatively correlated with Proteobacteria, Firmicutes, Bacteroidota, Chloroflexi, and Actinobacteriota (r = −0.984 to −0.905, *p* < 0.05). The highest positive correlation coefficient was observed between Proteobacteria and Ciliophora (r = 0.992, *p* = 0.0001), whereas the strongest negative correlation was detected between Proteobacteria and Protalveolata (r = −0.984, *p* = 0.0004).

## 4. Discussion

The gut bacterial microbiota of Nile tilapia was dominated by Fusobacteriota and Firmicutes, which together represented 89.54 ± 0.53% of the bacterial community in control fish and 86.23 ± 0.96% in infected fish. This pattern is consistent with previous studies in fish, where both phyla have been described as recurrent and dominant components of intestinal communities in aquaculture species, including cichlids such as tilapia [7]. However, DESeq2 identified infection-associated differences in the abundance of several bacterial phyla, including higher relative abundances of Proteobacteria and Firmicutes and lower relative abundances of Fusobacteriota, Patescibacteria, and Actinobacteriota. These taxon-specific findings should be interpreted cautiously considering the limited number of independent aquarium-level samples.

The differences observed in Fusobacteriota and Patescibacteria were consistent with the pattern previously reported in tilapia infected with *Providencia vermicola*, although the magnitudes differed. In the present study, the relative abundance of Fusobacteriota decreased from 76.62 ± 0.62% in control fish to 60.53 ± 4.60% in fish infected with *S. haemolyticus*, corresponding to a 21.0% reduction relative to the control. By comparison, *P. vermicola* infection reduced Fusobacteriota from 76.55 ± 0.67% to 40.54 ± 0.35%, representing a 47.0% reduction relative to the control [26]. Similarly, Patescibacteria decreased from 0.993 ± 0.078% to 0.093 ± 0.031% following *S. haemolyticus* infection, corresponding to a 90.6% reduction, whereas it was not detected above the filtering threshold in fish infected with *P. vermicola*. The shared direction of these changes is compatible with a broader microbiota response to infection-associated physiological stress or disruption of the intestinal environment. Nevertheless, the differences in magnitude suggest that pathogen-dependent effects may also contribute to the resulting microbial profiles. Because the two infections were evaluated in separate experiments, this comparison should be considered descriptive.

The increase in Proteobacteria is particularly relevant because the expansion of this phylum has been proposed as a microbial signature of intestinal dysbiosis, loss of community balance, inflammation, and increased disease risk [27]. Moreover, inflammation-driven dysbiosis models propose that an altered intestinal microenvironment may favor opportunistic or facultatively anaerobic bacteria, while reducing symbionts sensitive to inflammatory stress [28]. In fish, the gut microbiota contributes to epithelial barrier protection, immune modulation, and the exclusion of opportunistic pathogens; therefore, marked alterations in its composition may be associated with increased susceptibility to infectious outbreaks in aquaculture [7]. In this context, the higher relative abundance of Proteobacteria in infected fish was consistent with a microbial profile commonly associated with intestinal disturbance.

The decrease in Fusobacteriota was also relevant, especially because this phylum is commonly represented by *Cetobacterium* in freshwater fish [29]. In tilapia, *Cetobacterium* has been reported as a dominant or core component of the gut microbiota [30], and members of this genus have been associated with vitamin B12 production in the intestinal tract [31]. Moreover, *Cetobacterium somerae* has been shown to strengthen interactions within the microbiota, improve intestinal barrier integrity, and enhance host resistance to bacterial infections by producing vitamin B12 [32]. Therefore, the reduction in *Cetobacterium* in infected fish may indicate a decrease in bacterial groups that potentially contribute to metabolism, ecological stability, and intestinal defense.

Beyond the reduction in *Cetobacterium*, the genus-level analysis also showed decreases in *Romboutsia*, *Iamia*, *Dietzia*, *Terrimicrobium*, *Nordella*, and *Nocardioides* in fish infected with *S. haemolyticus*. Among these genera, the decrease in *Romboutsia* may have functional relevance. This genus has been reported as dominant in some Nile tilapia populations [33], and the family Peptostreptococcaceae, to which it belongs, has been associated with the fermentative metabolism of proteinaceous substrates and carbohydrates [34]. Thus, the reduction in *Romboutsia* may suggest a decrease in fermentative bacteria associated with nutrient utilization. In contrast, the decreases in *Iamia*, *Dietzia*, *Terrimicrobium*, *Nordella*, and *Nocardioides* should be interpreted with caution because their intestinal functions in tilapia are not clearly established. Their reduction may indicate broader community reorganization rather than direct evidence for the depletion of functionally beneficial bacteria.

Among the genera enriched in infected fish, the rise in *Staphylococcus* was compatible with the experimental challenge with *S. haemolyticus*. This pathogen has been described as an emerging agent in tilapia and has been shown to alter the gastrointestinal microbiota, generating signs of dysbiosis [11]. Therefore, the increase in *Staphylococcus* can be considered taxonomic evidence consistent with the establishment of the infectious process. In addition, the increase in *Mycobacterium* is relevant because several species within this genus are recognized as opportunistic pathogens of fish. *Mycobacterium marinum*, for example, can infect both freshwater and marine fish, causing granulomatous lesions and chronic disease [35]. Fish mycobacteriosis is also important in aquaculture systems, where it may be associated with morbidity, mortality, and unfavorable management conditions [36]. Therefore, the increase in *Mycobacterium* suggests that the intestinal environment of infected fish may have favored the expansion of potentially opportunistic bacterial groups.

The microeukaryotic community also differed between control and infected fish, indicating that the intestinal response was not restricted to the bacterial fraction. The intestinal microeukaryome of fish may include fungi, protists, microscopic metazoans, parasites, and transient organisms derived from food or the aquatic environment; therefore, its interpretation requires consideration of both host condition and culture-system ecology [4,37]. In infected fish, Ciliophora and Ascomycota had significantly higher relative abundances, whereas Nematozoa had a significantly lower relative abundance. Ciliates are functionally relevant protists in aquatic environments and participate in microbial trophic networks by consuming bacteria, algae, and other microorganisms [38]. Thus, the increase in Ciliophora may be related to greater availability of bacteria, detritus, or organic material during intestinal disturbance. Similarly, the increase in Ascomycota suggests a modification of the fungal component. In fish, Ascomycota has been reported as a frequent phylum of the gut mycobiota, and fungi may participate in digestive processes, microbial competition, immune stimulation, or protection against pathogenic bacteria [39]. However, in an infectious context, the increase in Ascomycota should be interpreted cautiously, as it may represent either a compensatory response or an opportunistic expansion favored by altered intestinal conditions.

The reduction in Nematozoa in infected fish could be associated with changes in feeding activity and intestinal content during infection. In fish, nematodes may occur as adult or larval endoparasites in the gastrointestinal tract, body cavity, or tissues, and some species use fish as intermediate, paratenic, or definitive hosts [40]. However, their detection in intestinal contents may also correspond to transient forms, remains of ingested organisms, or environmental components associated with the diet [4]. A reduction in Nematozoa was also observed in tilapia infected with *P. vermicola*, suggesting that this group may be sensitive to general changes associated with the infectious state, such as reduced feeding activity, altered intestinal transit, or changes in luminal content [26]. Therefore, the reduction in Nematozoa in fish infected with *S. haemolyticus* probably reflects an indirect response to the infectious state rather than a direct effect of the pathogen on this taxonomic group.

Together, the alpha- and beta-diversity results indicate that the differences between control and infected fish were primarily taxon-specific. Most beta-diversity comparisons did not detect significant differences between infected and control fish. Alpha diversity was higher in both bacterial and microeukaryotic communities, with higher Shannon and Simpson indices in infected fish. However, this increase should not be interpreted automatically as evidence of improved intestinal stability or host condition. Although higher diversity may enhance ecosystem functioning in some contexts, this relationship is not universal in animal microbiota [41,42,43]. Alpha diversity alone is insufficient to determine whether a microbiota is healthy, because high diversity may be beneficial, neutral, or detrimental depending on the host, community type, and physiological context [43]. Thus, the increase in diversity observed in infected fish may reflect a loss of dominance by resident taxa and the expansion of minor, opportunistic, or transient microorganisms favored by the infection-associated intestinal environment.

Beta-diversity analyses provided mixed evidence of infection-associated community variation. In bacteria, PERMANOVA did not detect significant differences using Bray–Curtis or Jaccard distances, whereas MiRKAT identified significant associations with both metrics. However, the small number of independent samples restricted the number of unique permutations and consequently limited the *p*-value resolution and statistical power of PERMANOVA. Therefore, the nonsignificant PERMANOVA results should not be interpreted as evidence that meaningful community differences were absent, but rather as inconclusive findings under the present level of replication. Because Bray–Curtis reflects abundance differences and Jaccard captures presence–absence variation, the MiRKAT results may indicate limited compositional variation associated with infection; however, the lack of agreement with PERMANOVA prevents a conclusive interpretation of community-level differences [44,45,46]. For microeukaryotes, most distance-based tests were not significant, providing no consistent evidence of broad community restructuring. The significant MiRKAT result obtained with weighted UniFrac may reflect more restricted variation in the relative abundance of phylogenetically related lineages, but this isolated result should be interpreted cautiously. Overall, the beta-diversity findings are compatible with selective or limited community changes rather than a generalized restructuring of the intestinal ecosystem.

The exploratory correlation analysis identified potential patterns of multi-kingdom co-occurrence. Proteobacteria, Firmicutes, and Bacteroidota were positively correlated with Ciliophora and Ascomycota, whereas Holozoa, Nematozoa, and Protalveolata were negatively correlated with Proteobacteria, Firmicutes, Bacteroidota, Chloroflexi, and Actinobacteriota. These patterns are consistent with the possibility that infection-associated changes in bacterial and microeukaryotic communities occurred concurrently; however, they do not demonstrate that the two microbial fractions responded in a coordinated or biologically linked manner. The positive correlations of Ciliophora with Proteobacteria, Firmicutes, and Bacteroidota could be compatible with trophic responses to bacterial availability, because ciliates are important bacterial consumers in aquatic microbial food webs [47,48]. Similarly, the correlations between Ascomycota and these bacterial phyla may reflect their concurrent responses to infection-associated changes in the intestinal environment. Conversely, the negative correlations involving Holozoa, Nematozoa, and Protalveolata may indicate contrasting patterns of variation between these microeukaryotic groups and the corresponding bacterial phyla. Nevertheless, because the network was constructed from only six independent microbiome samples, the high correlation coefficients should be interpreted exclusively as exploratory co-occurrence patterns. Such correlations may be influenced by individual observations, compositionality, sequencing depth, taxon rarity, limited replication, and treatment-associated covariation [49,50].

Predicted functional profiles suggested that infection was associated with differences in the functional potential of the gut bacterial microbiota. These results represent inferred metabolic potential rather than direct evidence of gene expression or metabolic activity because PICRUSt2 predicts functional profiles from 16S rRNA marker-gene data [22]. In the bacterial communities of infected fish, pathways predicted to have higher relative representation included peptidoglycan biosynthesis II (staphylococci), anhydromuropeptide recycling, L-arginine biosynthesis, arginine and polyamine biosynthesis, and fatty acid biosynthesis. The higher predicted representation of peptidoglycan biosynthesis and recycling pathways is biologically compatible with the infected condition because peptidoglycan is essential for cell-wall integrity in Gram-positive bacteria, while its recycling has been associated with bacterial survival under stationary-phase or stress conditions [51,52]. The predicted differences in arginine- and polyamine-related pathways may also be relevant because polyamines participate in bacterial growth, stress tolerance, host interactions, and survival strategies in several pathogens [53]. Likewise, the higher predicted representation of fatty acid biosynthesis pathways could indicate differences in the potential for bacterial membrane biosynthesis or modification, processes associated with stress responses in *Staphylococcus* spp. [54,55]. Nevertheless, these functional predictions require validation through direct approaches such as metagenomics, metatranscriptomics, proteomics, or metabolomics.

In contrast, control fish showed pathways mainly associated with heterolactic fermentation, the Bifidobacterium shunt, biotin biosynthesis, and cobalamin biosynthesis or salvage. These functions suggest a profile associated with fermentative metabolism and vitamin/cofactor metabolism. Because fermentative pathways can generate metabolites such as acetate and lactate that may be used within gut microbial networks, and vitamin-related pathways may provide essential cofactors for other community members, this profile may be compatible with metabolic exchange among gut microbes [32,56,57]. Cross-feeding interactions can contribute to the establishment, maintenance, and stability of gut microbial communities. Therefore, the control profile may reflect a microbial community with greater predicted potential for metabolic cooperation and intestinal homeostasis.

The thermal conditions used in this experiment provide an important context for interpreting the observed responses. Nile tilapia can tolerate 20 °C [58], but this temperature is below its commonly reported preferred temperature and optimal culture range [59]. Cooler conditions can modify physiological and immunological processes and may also influence the baseline configuration of the intestinal microbiota. Temperature was identical in control and infected aquaria and therefore cannot independently explain the differences between treatments; however, it may have influenced how the fish and their microbiota responded to *S. haemolyticus*. Consequently, the magnitude and characteristics of the infection-associated changes observed here may differ under the warmer conditions normally encountered in commercial tilapia farming. Evaluating temperature and infection simultaneously in future factorial experiments would help determine the extent of this context dependence.

This study has several limitations that should be considered when interpreting the results. Only three aquaria were used per treatment, and intestinal contents from three fish within each aquarium were pooled, resulting in three independent microbiota samples per treatment. Although this pooling strategy provided an aquarium-level composite sample and avoided treating fish maintained in the same aquarium as independent experimental replicates, it did not increase biological replication and prevented the assessment of interindividual variation. The limited number of independent samples may have reduced statistical power across the alpha-diversity, beta-diversity, differential-abundance, and correlation analyses, particularly for detecting small or moderate effects. DESeq2 was selected because its negative binomial framework is applicable to differential-abundance analysis of unrarefied microbial taxon counts [21,60]. However, under the present level of replication, its dispersion and effect-size estimates have limited precision. Consequently, significant differential-abundance findings should be interpreted cautiously, whereas nonsignificant results cannot exclude small or moderate biological differences. Confirmation using more independent aquaria and complementary differential-abundance methods is therefore warranted [61]. In addition, the observed associations between bacteria and microeukaryotes represent patterns of co-occurrence rather than evidence of direct biological interactions. PICRUSt2 profiles represent predicted functional potential, and their reliability depends on the phylogenetic proximity of the detected ASVs to sequenced and functionally annotated reference genomes. Limited taxonomic resolution and the incomplete genomic representation of fish-associated microbial lineages may therefore increase uncertainty in the inferred pathway profiles [23,62]. Future studies should include more independent aquaria, individual fish samples analyzed using an appropriate nested design, pre-challenge microbiota measurements, complementary differential-abundance methods, and direct functional approaches such as shotgun metagenomics, metatranscriptomics, proteomics, or metabolomics.

From an aquaculture-health perspective, these infection-associated microbiota differences highlight the relevance of management strategies that consider intestinal microbial balance, including the use of probiotics, prebiotics, and medicinal plant products as complementary approaches to disease control [63]. Overall, the results indicate that *S. haemolyticus* infection was associated with a multi-kingdom alteration in the intestinal microbiota in Nile tilapia. This alteration did not correspond to a complete replacement of the intestinal communities, but rather to a moderate ecological reorganization involving bacterial and microeukaryotic composition, diversity patterns, co-occurrence patterns, and predicted functional potential. However, the predicted functional profiles and co-occurrence patterns should be considered hypothesis-generating and require confirmation using greater biological replication and direct functional approaches.

## 5. Conclusions

This study indicates that experimental infection with *S. haemolyticus* was associated with taxon-specific differences in both the bacterial and microeukaryotic components of the intestinal microbiota of Nile tilapia. The results suggest that the infectious process was associated not only with differences in bacterial taxa compatible with the experimental challenge, but also with a moderate reorganization of the gut microbiota. This pattern involved shifts in microbial composition, diversity patterns, associations between bacterial and microeukaryotic groups, and predicted bacterial functional potential. The microbial differences associated with *S. haemolyticus* may have important implications for microbial stability, host–microbiota interactions, and intestinal health in farmed fish. Overall, the integrated analysis of bacterial and microeukaryotic communities and predicted functional profiles provides a more comprehensive view of the intestinal response to bacterial infection and underscores the need to consider the gut microbiota from a multi-kingdom perspective in aquaculture health studies.

## Figures and Tables

**Figure 1:**
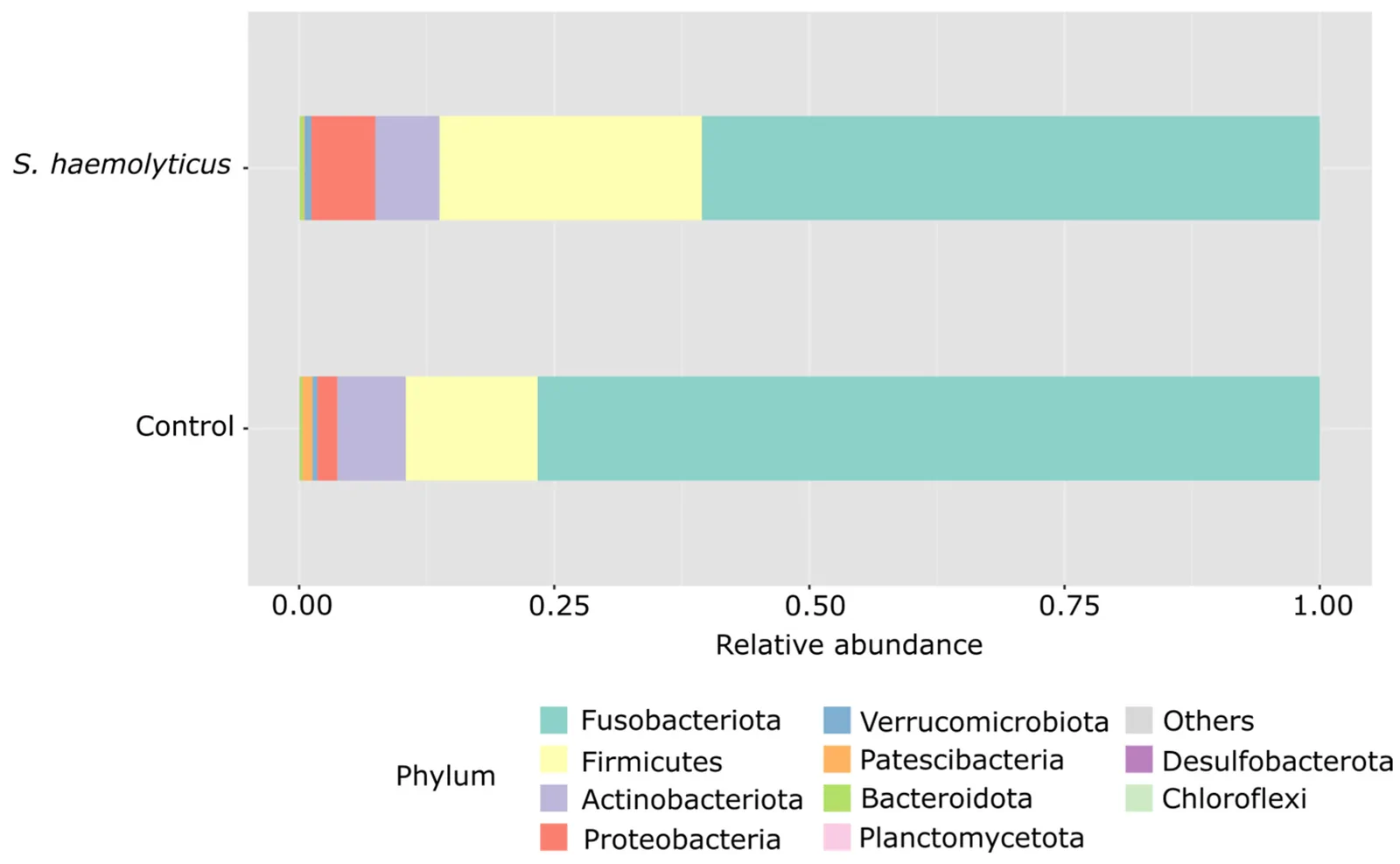
Stacked bar plot showing the relative abundance of bacterial phyla in the gut microbiota of control Nile tilapia (Control) and infected Nile tilapia (*S. haemolyticus*) (*n* = 3 pooled samples per treatment).

**Figure 2:**
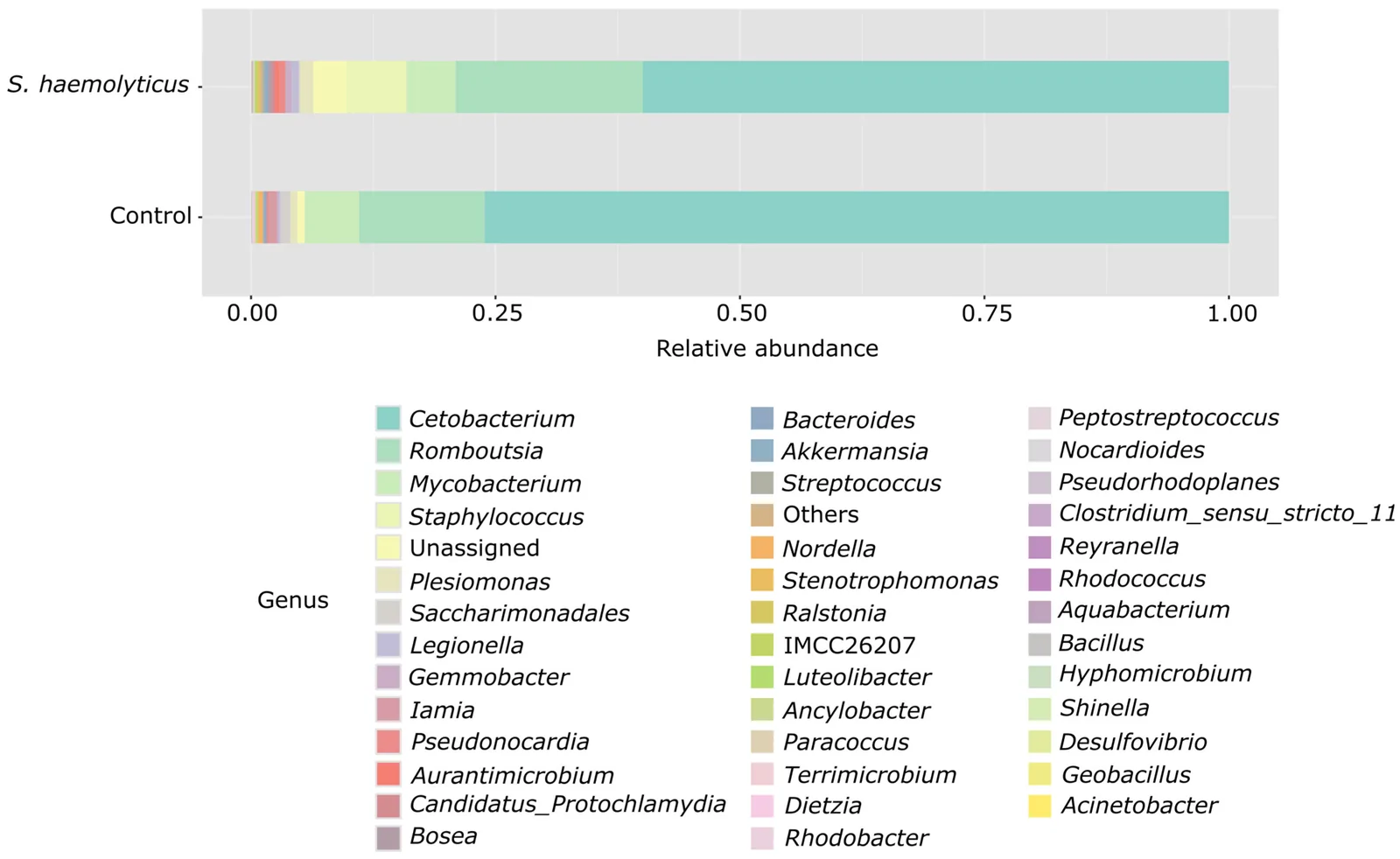
Stacked bar plot showing the relative abundance of bacterial genera in the gut microbiota of control Nile tilapia (Control) and infected Nile tilapia (*S. haemolyticus*) (*n* = 3 pooled samples per treatment).

**Figure 3:**
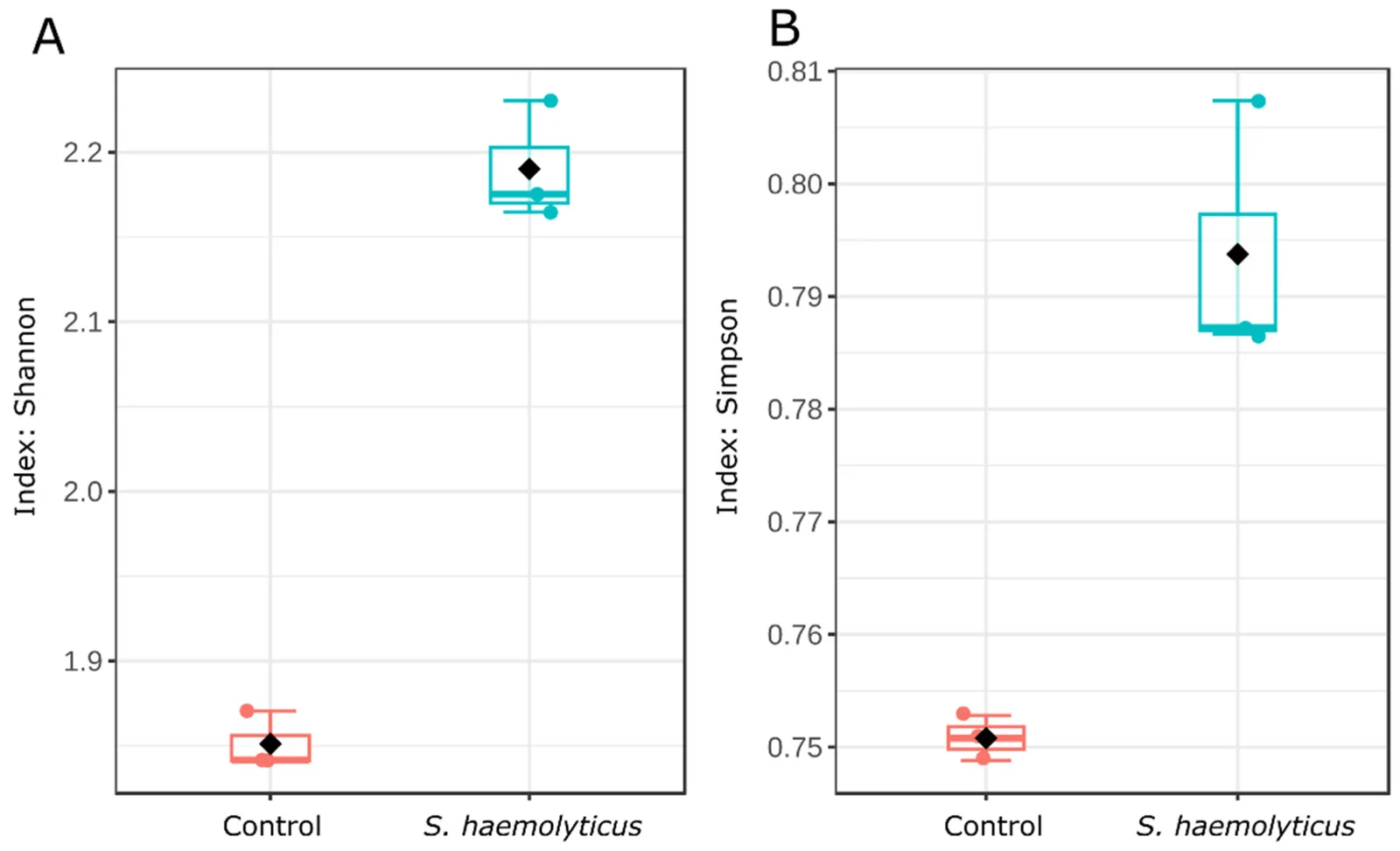
Alpha diversity of the gut bacterial microbiota based on the Shannon (**A**) and Simpson (**B**) indices at the ASV level in control Nile tilapia (Control) and infected Nile tilapia (*S. haemolyticus*). Dots represent individual samples, and diamonds indicate group means. Significant differences were established at *p* < 0.05.

**Figure 4:**
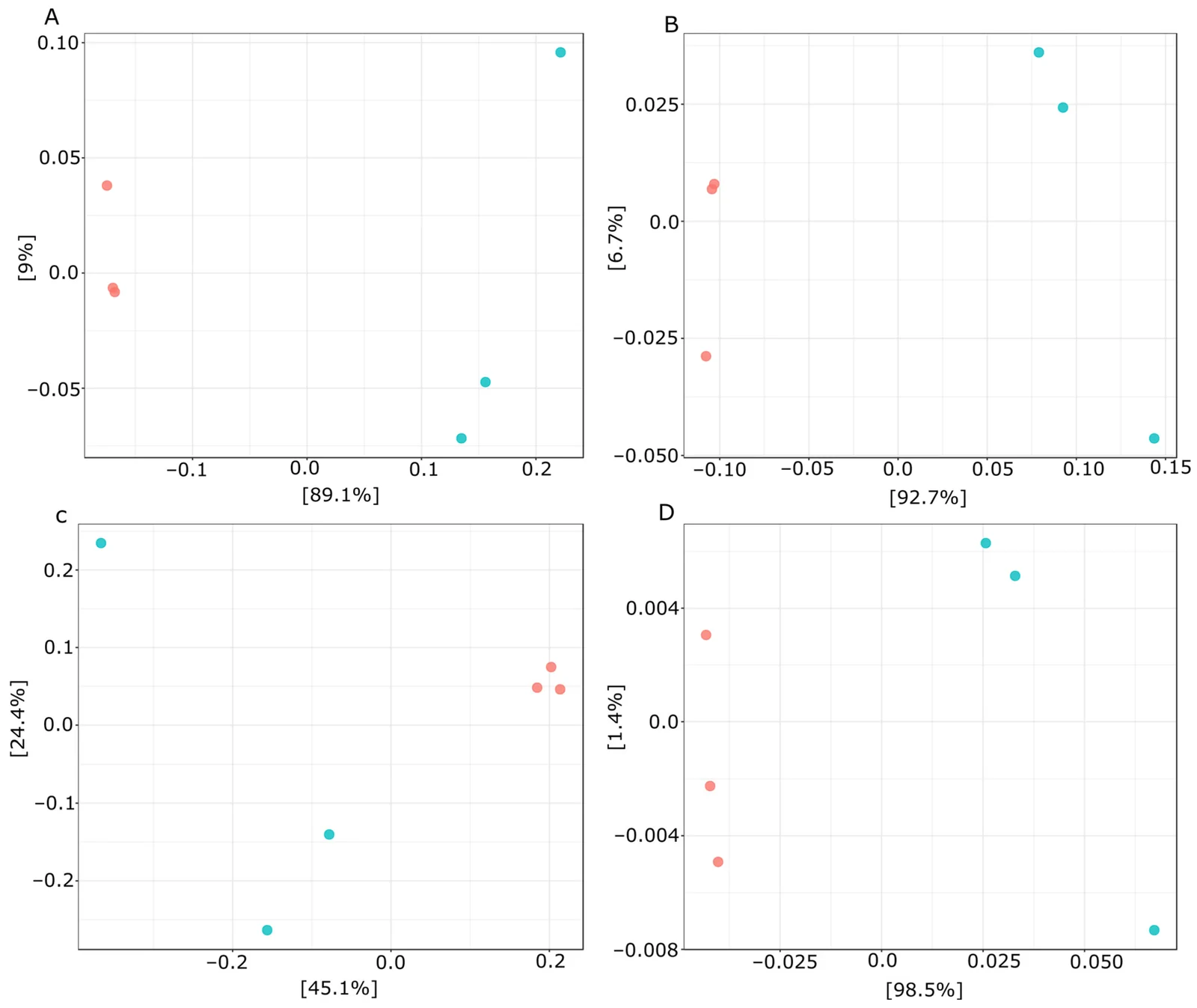
Principal coordinates analysis (PCoA) of the gut bacterial microbiota at the ASV level based on Jaccard (**A**), Bray–Curtis (**B**), unweighted UniFrac (**C**), and weighted UniFrac (**D**) distance metrics in control Nile tilapia and fish infected with *S. haemolyticus*. Red symbols indicate control samples, whereas blue symbols indicate samples from fish infected with *S. haemolyticus*. Statistical significance was evaluated using PERMANOVA and MiRKAT.

**Figure 5:**
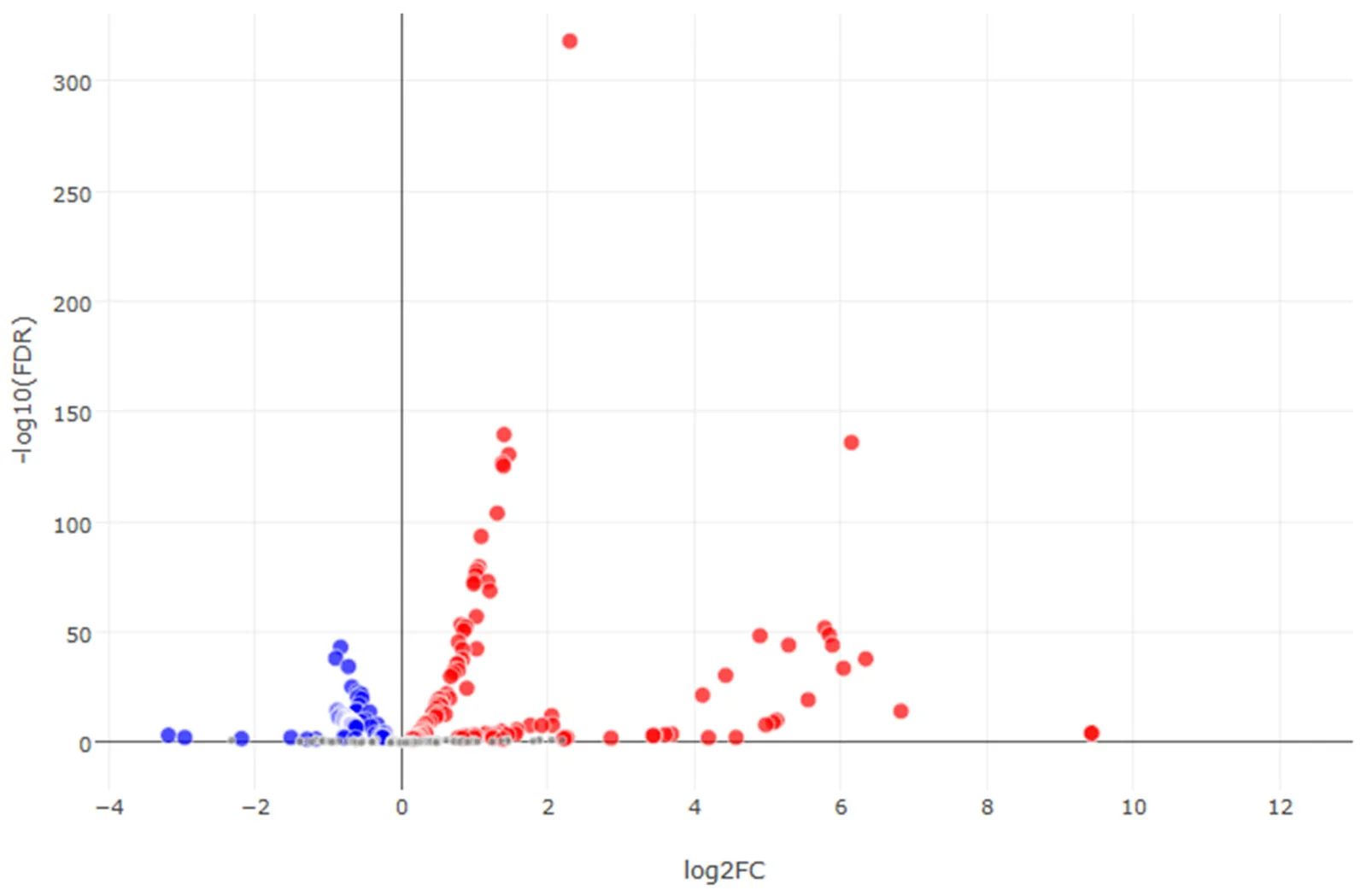
Volcano plot of differentially abundant predicted MetaCyc pathways in the gut bacterial microbiota of control Nile tilapia and fish infected with *S. haemolyticus*. Each point represents a predicted MetaCyc pathway. The *x*-axis indicates log_2_ fold change, and the *y*-axis indicates −log_10_(FDR). Blue points indicate pathways with higher predicted abundance in the control group, whereas red points indicate pathways with higher predicted abundance in fish infected with *S. haemolyticus*.

**Figure 6:**
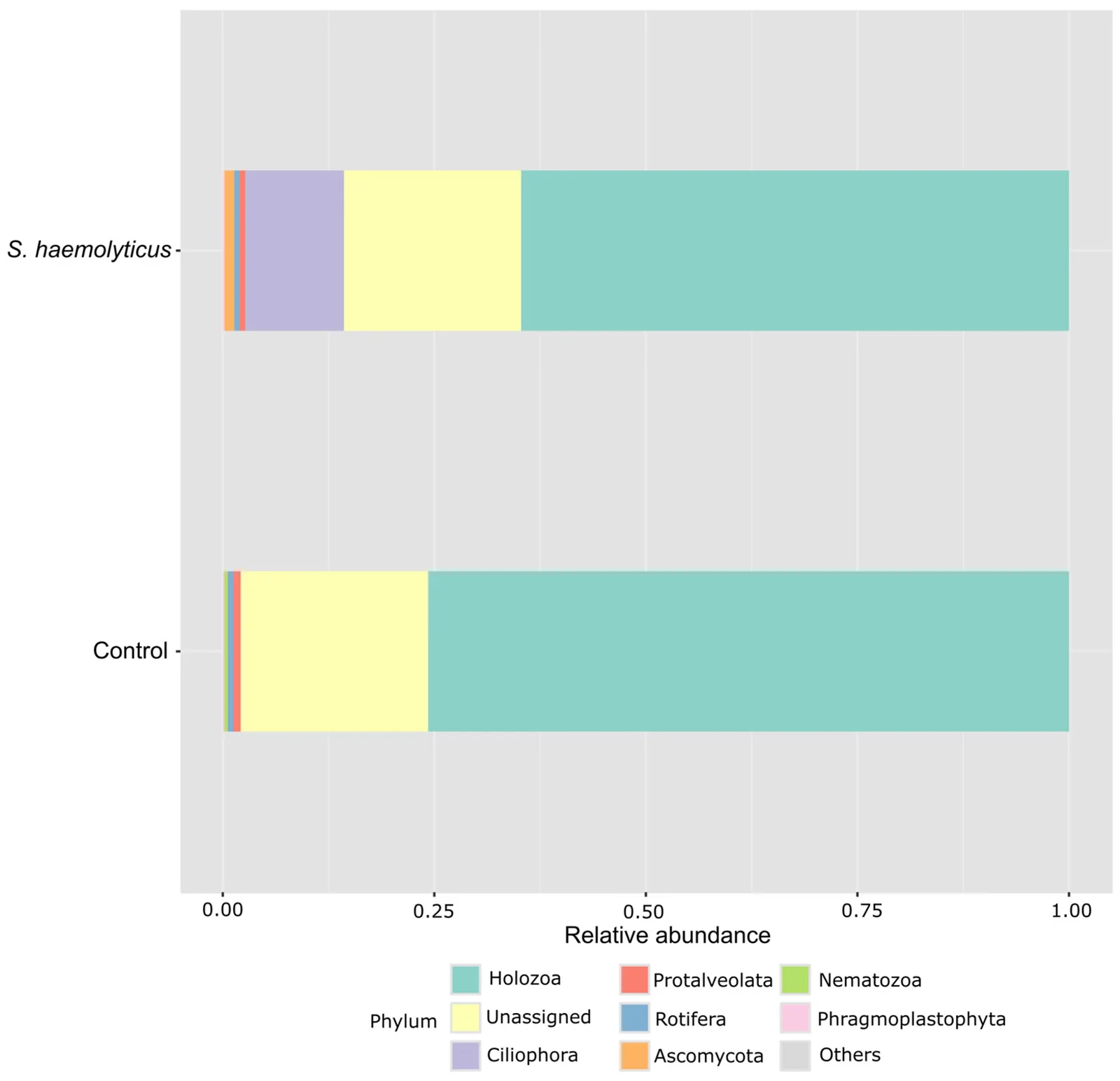
Stacked bar plot showing the relative abundance of microeukaryotic phyla of the gut microeukaryotic microbiota in control Nile tilapia (Control) and infected Nile tilapia (*S. haemolyticus)* (*n* = 3 pooled samples per treatment).

**Figure 7:**
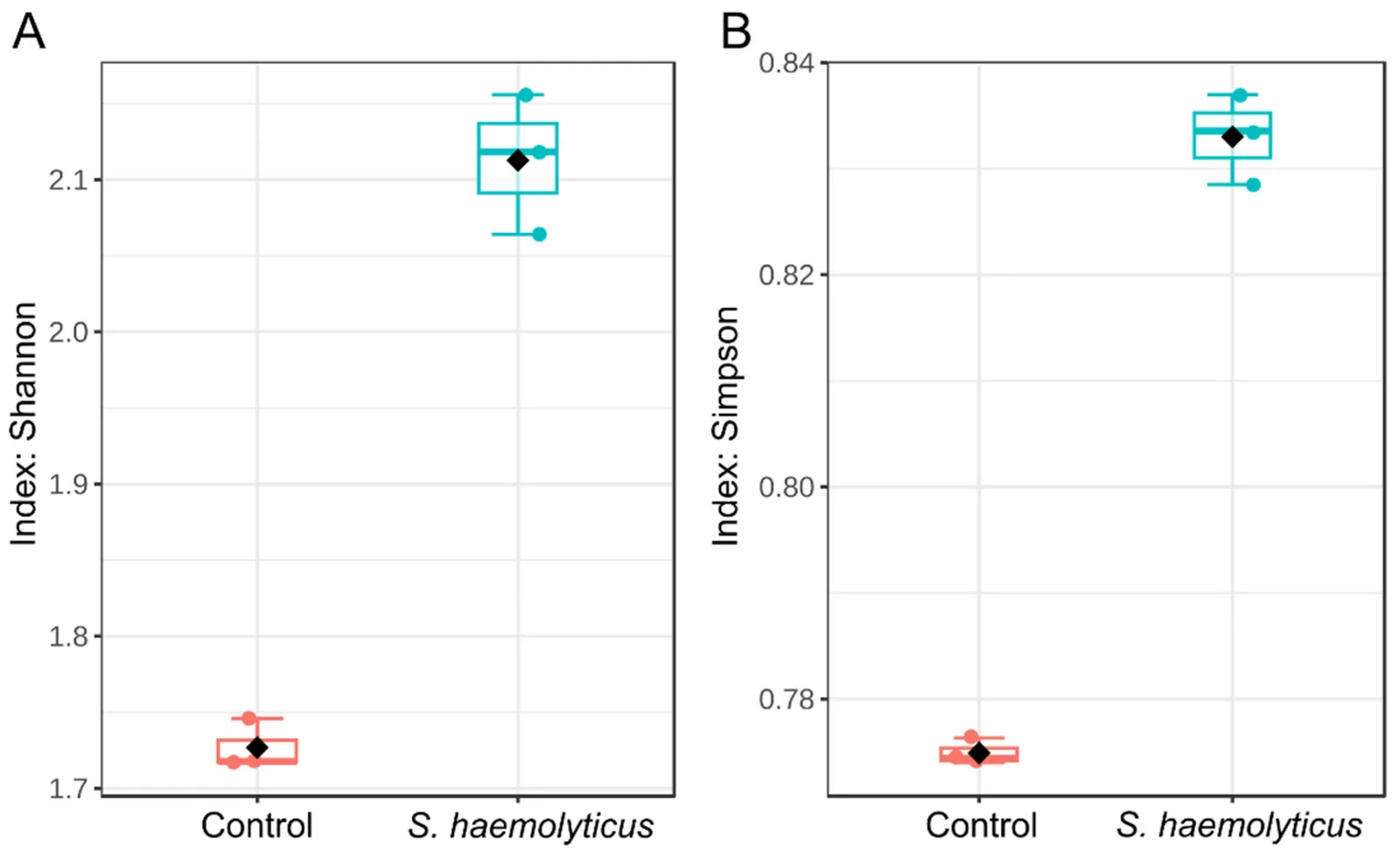
Alpha diversity of the gut microeukaryotic community based on the Shannon (**A**) and Simpson (**B**) indices at the ASV level in control Nile tilapia (Control) and infected Nile tilapia (*S. haemolyticus*). Dots represent individual samples, and diamonds indicate group means. Significant differences were established at *p* < 0.05.

**Figure 8:**
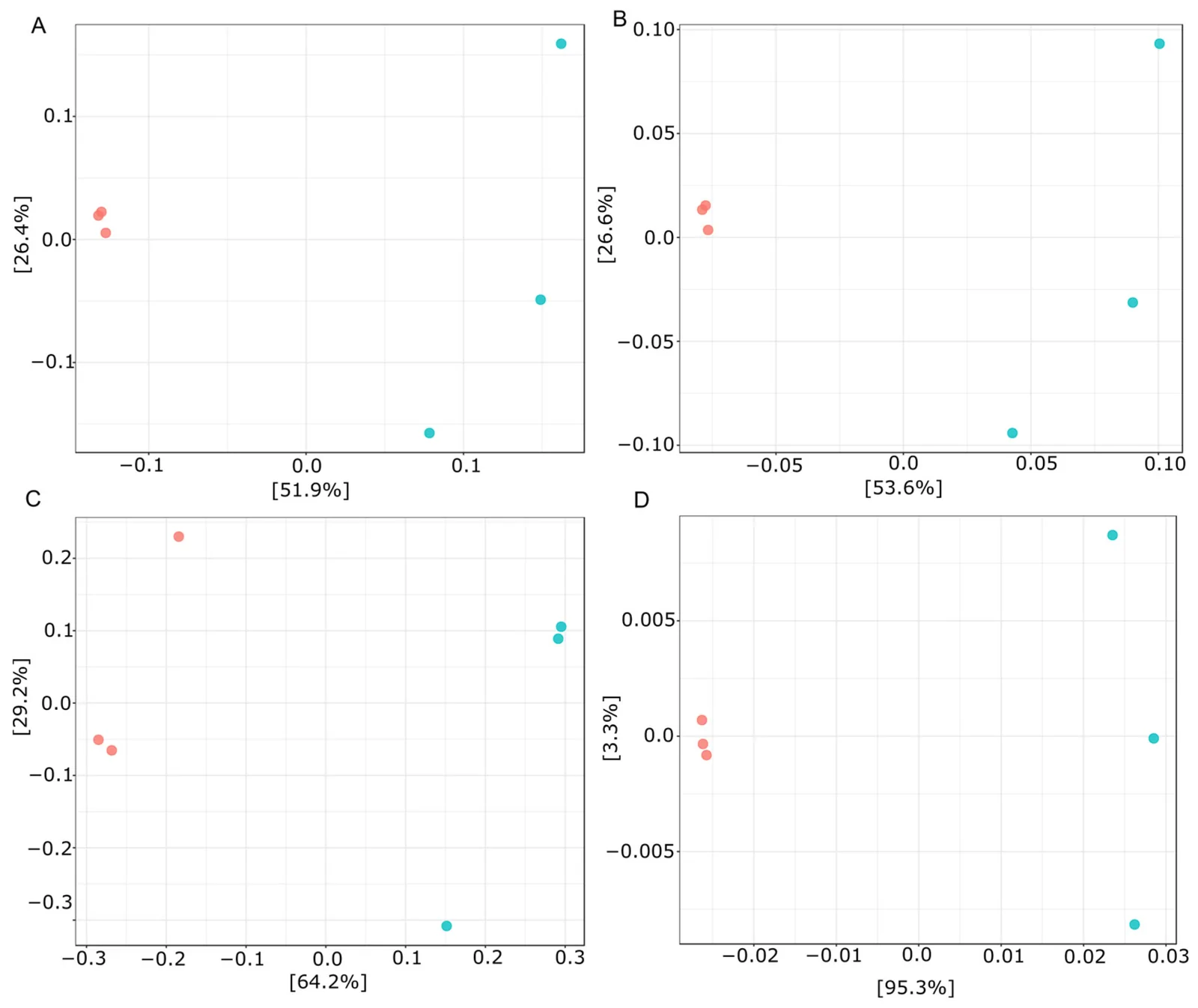
Principal coordinates analysis (PCoA) of the gut microeukaryotic community at the ASV level based on Jaccard (**A**), Bray–Curtis (**B**), unweighted UniFrac (**C**), and weighted UniFrac (**D**) distance metrics in control Nile tilapia and fish infected with *S. haemolyticus*. Red symbols indicate control samples, whereas blue symbols indicate samples from fish infected with *S. haemolyticus*. Statistical significance was evaluated using PERMANOVA and MiRKAT.

**Figure 9:**
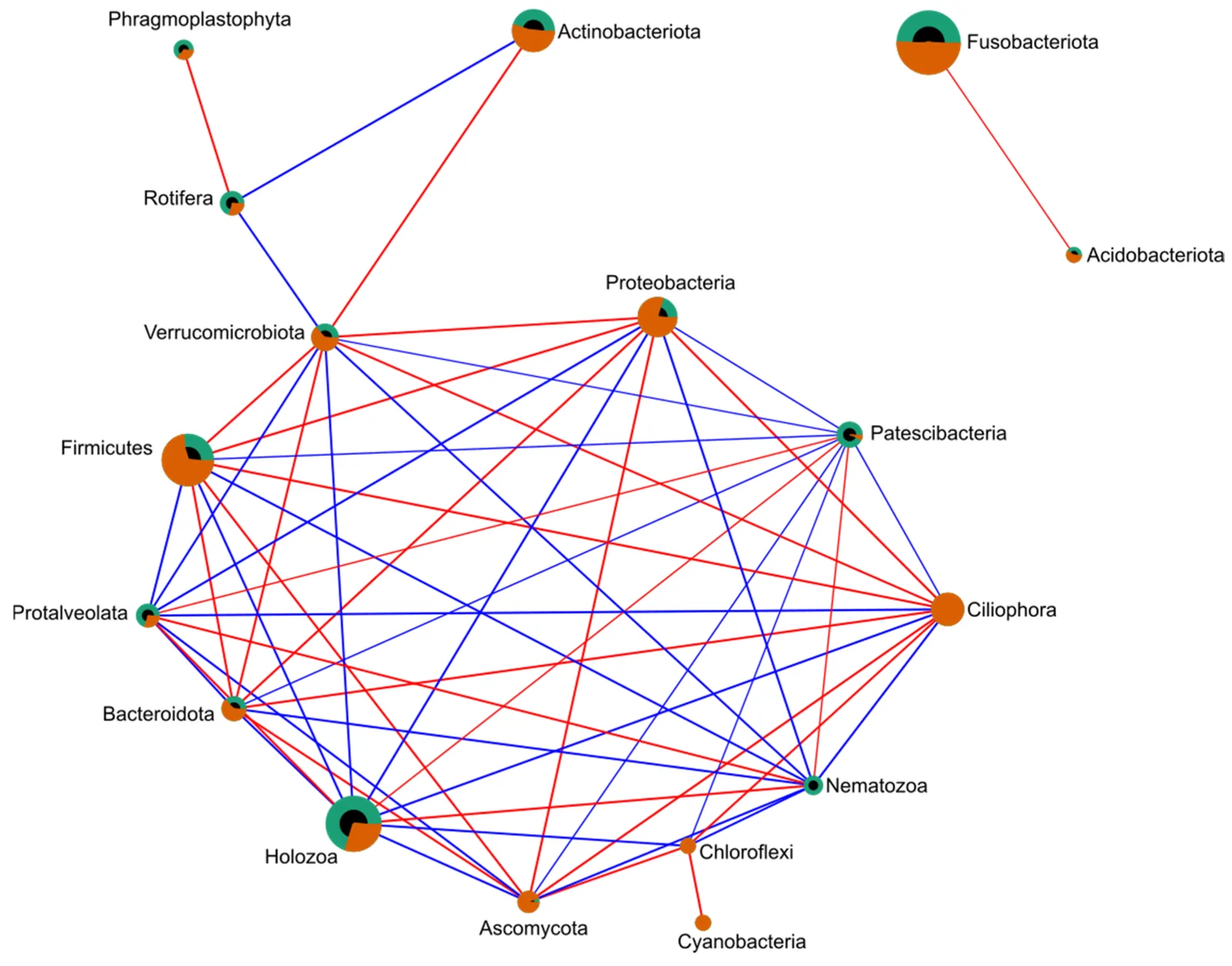
Pearson correlation-based co-occurrence network between bacterial phyla and comparable high-level microeukaryotic taxa in the gut microbiota of control Nile tilapia and fish infected with *S. haemolyticus*. Node color indicates the treatment in which each taxon showed higher relative abundance: green for the control group and orange for the infected group. Red edges indicate positive correlations (r > 0), whereas blue edges indicate negative correlations (r < 0). Only correlations with *p* < 0.05 are shown.

## Data Availability

The data presented in this study are available on request from the corresponding author.

## Supplementary Materials

The following supporting information can be downloaded at https://www.mdpi.com/article/10.3390/ani16192991/s1, Table S1: Water quality parameters measured in each experimental treatment; Table S2: Sequencing depth and rarefaction information for the 16S and 18S datasets; Table S3: Beta diversity statistical analysis of bacterial and microeukaryotic gut communities using PERMANOVA and MiRKAT.
