# Supplementary material for: Staphylococcus haemolyticus Infection Modifies Bacterial and Microeukaryotic Gut Communities in Nile Tilapia
Source: Animals (Basel). 2026 Sep 23;16(19):2991. doi: 10.3390/ani16192991 (PMC13663544; doi:10.3390/ani16192991)
Supplement: Supplementary file 1 [file animals-16-02991-s001.zip › animals-4559364-supplementary.pdf]

Supplementary Material

Supplementary Table S1. Water quality parameters measured in each experimental treatment.

Treatment	NH_4^+ (mg L^{-1})	NO_2^- (mg L^{-1})	NO_3^- (mg L^{-1})
Control	0.28 ± 0.21	0.68 ± 0.53	0.21 ± 0.16
S. haemolyticus	0.30 ± 0.21	0.79 ± 0.65	0.17 ± 0.15

Supplementary Table S2. Sequencing depth and rarefaction information for the 16S and 18S datasets

Marker	Sample ID	Treatment	Input read pairs	Sequences retained in the final filtered feature table	Rarefaction depth
16S	16S-1	Control	25,780	23,206	18,672
16S	16S-2	Control	38,353	34,307	18,672
16S	16S-3	Control	28,173	25,307	18,672
16S	16S-4	*S. haemolyticus*	26,225	23,420	18,672
16S	16S-5	*S. haemolyticus*	25,060	22,591	18,672
16S	16S-6	*S. haemolyticus*	21,170	18,672	18,672
18S	18S-1	Control	41,054	17,678	5,082
18S	18S-2	Control	36,482	15,680	5,082
18S	18S-3	Control	39,651	17,259	5,082
18S	18S-4	*S. haemolyticus*	32,396	5,462	5,082

18S	18S-5	*S. haemolyticus*	32,981	5,670	5,082
18S	18S-6	*S. haemolyticus*	31,335	5,082	5,082

Note: Input read pairs correspond to the sequences obtained before DADA2 processing. Final sequence counts correspond to the feature tables used for downstream analyses after quality filtering, denoising, paired-read merging, chimera removal, and applicable taxonomic filtering. For the 18S dataset, ASVs classified as Vertebrata were excluded before rarefaction. The rarefaction depth corresponds to the minimum sequence count in the final feature table for each marker.

Supplementary Table S3. Beta diversity statistical analysis of bacterial and microeukaryotic gut communities using PERMANOVA and MiRKAT.

Microbiota fraction	Distance metric	PERMANOVA pseudo-F	PERMANOVA R^2	PERMANOVA p-value	PERMANOVA FDR	MiRKAT R^2	MiRKAT p-value	MiRKAT KRV
Bacterial	Bray-Curtis	34.99	0.89	0.1	0.1	0.93	0.004	2.8
Bacterial	Jaccard	27.31	0.87	0.1	0.1	0.94	0.0009	1.08
Bacterial	Unweighted UniFrac distance	2.83	0.41	0.1	0.1	0.58	0.07	0.44
Bacterial	Weighted UniFrac distance	36.68	0.90	0.1	0.1	0.83	0.08	15.87
Microeukaryotic	Bray-Curtis	4.35	0.52	0.1	0.1	0.70	0.1	3.78

Microeukaryotic	Jaccard	4.14	0.50	0.1	0.1	0.69	0.1	1.34
Microeukaryotic	Unweighted UniFrac distance	6.63	0.62	0.1	0.1	0.77	0.13	0.41
Microeukaryotic	Weighted UniFrac distance	75.37	0.94	0.1	0.1	0.99	0.001	48.66

Note: PERMANOVA F, pseudo-F statistic; PERMANOVA R^2, proportion of variation in the distance matrix explained by treatment; PERMANOVA p-value, unadjusted statistical significance value; PERMANOVA FDR, false discovery rate-adjusted p-value; MiRKAT R^2, proportion of microbiome variation associated with treatment under the MiRKAT framework; MiRKAT p-value, unadjusted statistical significance value; MiRKAT KRV, kernel RV statistic measuring the association between treatment and microbiota community structure. $p < 0.05$ was considered statistically significant.
